# A qualitative analysis of women’s reproductive agency and postpartum family planning in Maharashtra, India

**DOI:** 10.1371/journal.pone.0336103

**Published:** 2025-11-07

**Authors:** Nandita Bhan, Edwin Elizabeth Thomas, Gennifer Kully, Mohan Ghule, Anita Raj, Lotus McDougal, Abhishek Singh, Sarah Averbach

**Affiliations:** 1 Jindal School of Public Health and Human Development, O.P. Jindal Global University, Sonipat, Haryana, India; 2 Center on Gender Equity and Health, University of California San Diego, La Jolla, California, United States of America; 3 Department of Gender and Women’s Studies, University of Wisconsin, Madison, Wisconsin, United States of America; 4 Department of Obstetrics, Gynaecology and Reproductive Sciences, University of California, San Diego, La Jolla, California, United States of America; 5 Newcomb Institute, Tulane University, New Orleans, Louisiana, United States of America; 6 Celia Scott Weatherhead School of Public Health and Tropical Medicine, Tulane University, New Orleans, Louisiana, United States of America; 7 Department of Public Health & Mortality Studies and Centre of Demography of Gender, International Institute for Population Sciences, Mumbai, Maharashtra, India; TERI School of Advanced Studies, INDIA

## Abstract

**Background:**

Despite the benefits of healthy birth spacing for mothers and infants, the use of postpartum family planning (PPFP) by women in India remains low. We qualitatively examined barriers to PPFP access and use to understand the intersections between women’s reproductive agency, fertility and contraceptive norms, and community interactions with health providers in rural Maharashtra, India.

**Methods:**

We conducted 62 qualitative in-depth interviews with postpartum women, husbands and mothers-in-law of postpartum women, frontline health workers (FLWs) and key community stakeholders in rural Maharashtra from March to May 2022. Semi-structured interview guides included probes related to knowledge of and access to PPFP services, contraceptive decision-making dynamics, interactions with health providers, community norms related to fertility and family planning, and existing and aspirational models for PPFP service delivery. We used an inductive approach to organize emerging codes into themes using Bronfenbrenner’s Ecological Systems Theory, categorizing findings into a thematic framework to inform PPFP programming.

**Results:**

Five key themes emerged: (1) Few women received PPFP counseling or services, with marginalized communities disproportionately affected by poor access. (2) Postpartum women lacked agency in contraceptive decision-making and often needed their husband’s approval to access PPFP services. (3) Clinicians identified misinformation, lack of education, and community beliefs in contraceptive myths for lack of informed choice. (4) While norms for limiting family size were strong, contraception was considered a ‘sensitive’ subject and sparked concerns regarding confidentiality in discussing PPFP. (5) Community reported that counseling services were prescriptive and lacked information on the side effects of contraception, leading to the perception of low-quality care and dissatisfaction with PPFP health services.

**Conclusions:**

There is an urgent need to enhance PPFP counseling and service provision within family planning programs in India, emphasize reproductive choice among women, broaden the public dialogue on contraceptive use and find ways to engage men in contraceptive decision making. Interventions that can enhance knowledge and change acceptability of contraception in the community will enhance informed choice for contraceptive use among couples as well as PPFP access and use.

## Introduction

The health and nutrition benefits of healthy birth spacing for women and children are well documented worldwide [1–7]. Use of postpartum contraception [8] supports optimum inter-pregnancy intervals, leading to intergenerational benefits and helping to prevent some of the adverse effects of short birth spacing [9–11]. In low-and-middle-income countries (LMICs) like India, with the continued burden of undernutrition and significant maternal and child morbidity, postpartum contraception offers multiple benefits for women and children [10,12]. However, there has been a notable gap in our understanding of women’s reproductive health service needs, access and experiences in the postpartum period with implications for the delivery of postpartum family planning (PPFP) counseling and services. In India, family planning (FP) programs and interventions have often focused on delaying early fertility as well as limiting family size. In this context, the barriers to women’s access to postpartum contraception are poorly understood.

In recent years, there have been a notable decline in birthrates in India accompanied by a steady rise in the use of modern contraception, which is reflected by improvements in indicators such as the total fertility rate (TFR) and modern contraceptive prevalence rate (MCPR) [13]. These improvements have often been attributed to the promotion of female permanent contraception, with one estimate showing that nearly a third of female permanent contraception in public institutions were completed in the postpartum period [14]. The prevalence of reversible contraception use in the postpartum period India is low [15]. An analysis of data from the 2015–16 National Family Health Surveys (NFHS) shows that less than one in ten Indian women (9%) used modern contraception one month after delivery, increasing to only one in five (21%) at six months and one in four (26%) at twelve months [16]. In rural Maharashtra, among women who delivered a baby within the past 12 months and used postpartum family planning within three months of that delivery, the most common form of postpartum family planning was female permanent contraception (39%), followed by condoms (34%) and intrauterine devices (IUDs) (10%) (authors’ calculations from 2019–21 NFHS-5 data) [13]. These data must be interpreted in light of the national indicators where 56.5% (rural: 55.5% and urban: 58.5%) of currently married women 15–49 years reported using any modern contraception with IUD/immediate postpartum IUD (PPIUD) use at 2.1%. Maharashtra, where this study was conducted reports a higher than national average modern contraception prevalence of 63.8% (rural: 64.7%, urban: 62.7%) in 2019–2021, with IUD/PPIUD prevalence of 1.9%. While condoms and IUDs been the most common reversible methods used during the postpartum period, methods such as injectable contraception and progestin-only pills (POP) remain rare. IUDs have been one of the oldest long-acting reversible contraceptive method used in the Indian public health system. Immediate postpartum IUD (PPIUD) use is often promoted within primary health systems due to the high effectiveness and the perception of ease of use postpartum. Despite this, the uptake of IUDs in the Indian context has been limited, mainly attributed to myths and side effects related to their use, and women’s dissatisfaction, which are often not well understood [17].

The role of women’s reproductive agency and choice as well as FP norms as influencers of postpartum FP access and use remains poorly understood. Reproductive autonomy has been defined as, “having the power to decide about and control matters associated with contraceptive use, pregnancy and childbearing.” [18]. Reproductive agency - – the act of working towards one’s self-determined reproductive goals – situates this idea within the contextual factors that influence that power and can be represented by a cyclical interaction of Can-Act-Resist [19] cyclical interaction of that reflects a relational view of the exercise of women’s choice. This agency can be critical in determining PPFP access and use, navigating the expectations and pressures of significant others and in women’s recovery as well as caregiving for the newborn. Additionally, there is limited understanding of the norms related to ideal family size, son preference and childbearing that influence the decision to use FP, and in particular PPFP. These gaps relate not only to barriers in the use of contraception, but also its understanding determinants related to fertility, childbearing and son preference that influence family planning choices and behaviors [20–22]. PPFP programs often operate within the context of restrictive gender norms, compromised agency with regards to the resumption of sexual activity, social preferences for at least one male child, social isolation in the postpartum period, partner family control over mobility, and an understaffed and fragmented health system [23–25]. These aspects at the level of individuals, families and communities have not been understood in previous studies. There are limited data exploring the intersections of these complex determinants. This context can be challenging and as a result, many FP programs continue to deliver contraception using outmoded practices or platforms without recognizing women’s needs and desires for family planning services, leading to limited outreach to women who have recently delivered, and who need contraception the most. Additional barriers faced include low women’s agency, lack of participation in family planning decision-making, and norms that do not encourage conversation and participation in FP by women.

Current global evidence, largely drawn from quantitative studies, finds a range of agency-related aspects influencing PPFP, including women’s education and their autonomy, spousal communication and decision-making for use or non-use, social support, and to a lesser extent, women’s occupation and lack of correct knowledge on family planning limiting women from exercising their choice and leading to fear of side effects [26–33]. Evidence from Ghana among postpartum women indicates their agency in the discussion on family planning with their male partner, but they still require their partner’s approval to use modern contraception [26]. In Kenya, women’s agency within the context of PPFP also manifested in covert use; women who opted for PPFP despite their partner’s disapproval feared that contraceptive side effects might expose their use [32].

We conducted a qualitative study to gain a deeper understanding of how agency and gender norms influence contraceptive use in India. This study was part of a larger project examining the facilitators and barriers to PPFP. We focused on evaluating the feasibility of integrating family planning into other health services, with particular focus on women’s agency and wider social norms related to postpartum contraception used for birth spacing. The study was conducted in Maharashtra where levels of female education have been rising, noted in the percentage of women with 10 or more years of schooling increasing from 42% in 2015−16 to 50% in 2019−21 [34]. Our analyses focused on identifying postpartum women’s need for contraception and their agency to meet these needs, the role of norms guiding PPFP access and use, and community engagement with PPFP services.

## Methods

### Study design and setting

We conducted qualitative interviews as part of a study to understand the feasibility of delivering PPFP services through infant vaccination camps offered by the public health system in India. Vaccination camps, delivering routine immunization, comprised public delivery points for child health services and could be utilized for greater outreach for family planning, particularly postpartum family planning counseling and service delivery. These immunization camps could serve as a platform for delivering family planning services, overcoming barriers related to outreach and health service access towards young mothers.

These interviews were conducted between March and May 2022 in Maharashtra, India. Maharashtra is a rapidly urbanizing state that has also seen an expansion of health services. The proportion of women using contraception in 2019−21 was similar to the national average; the unmet need for family planning was higher than the national average [34]. The state has higher rates of female permanent contraception (44%) than the national average, while the rates of PPIUD are lower than the national average [34]. These use patterns provide a unique setting for understanding the low use of reversible postpartum contraception.

### Sample and recruitment

We conducted one-on-one interviews with a sample of 62 participants including: women 12 weeks postpartum or less (n = 22), husbands of postpartum women (n = 10), mothers-in-law (MIL) of postpartum women (n = 10), frontline health providers (n = 10), and village leaders (n = 10). Eligible postpartum women, husbands and MILs were recruited by a gender-matched research assistant near antenatal clinics, primary health centers, community centers and immunization camps. We did not seek to recruit eligible participants from the same family, nor did we attempt to link data between family members. More postpartum women were interviewed given the need to center the lived experience of these women in this work. However, the study team also explored the perspectives of other important stakeholders including 10 interviews each among frontline healthcare workers and community leaders (including NGO leadership and primary health center medical officers). Health providers were recruited at their place of work. Community leader participants were asked for referrals to other potential participants, as needed, to reach the desired sample size. Interviews were conducted in households and at health centers per the convenience of participants and lasted for 40 minutes.

Data were collected via semi-structured interviews that were developed based on the study objectives and research literature identified by investigators. All interviews were conducted in the local language (Marathi). Data were collected by four Master’s degree-level field investigators who were trained in qualitative data collection, had local language expertise and understood the nuances of local cultural practices. Our research followed ethical norms and standards as per the norms for research. All mandated protocols were followed for data collection and management. Written informed consent was obtained from the study participants. None of the postpartum women who participated in the study were minors or non-literate. Three mothers-in-law, ages 50–60 years who participated in the study were non-literate. Interviews were audio-recorded with the consent of the participant and subsequently transcribed and translated into English. For participants with limited or no literacy, informed consent was obtained in front of a literate witness, who also had the opportunity to ask questions.

Interviews were audio-recorded with the consent of the participant and subsequently transcribed and translated into English. The study received ethical approval from the Human Research Protections Program at University of California San Diego in the United States, and the Sigma Institutional Review Board (A Division of Sigma Research and Consulting Pvt Ltd) in India.

### Interviews probes

Our study utilized Urie Bronfenbrenner’s *Ecological Systems Theory* to identify stakeholders as well as their spheres of influence [35]. Bronfenbrenner’s Ecological Systems Theory, originally theorized to understand child development [35], was used to define and frame the interconnected system of influencers who guided women’s agency and choice. Levels within the theory were used to define women’s postpartum contraceptive use as being determined by women’s own agency for use and the role of key family members -husbands and mothers-in-law (family agents), community health workers (institutional agents) and local leaders and elected officials (community agents). This conceptual framework guided the thematic areas of investigation, instrument development, analysis and the synthesis of findings.

Interview probes with *postpartum women* (n = 20) focused on: a) postpartum counseling during antenatal care and delivery and PPFP services; b) issues related to women’s agency in the uptake of PPFP and factors that may improve agency and PPFP; c) knowledge of PPFP services, decision-making preferences and any experience of family or provider coercion; d) norms affecting contraceptive use and expectations related to fertility/childbearing and around spacing, son preference and specific contraceptive types; e) feasibility and challenges related to community based FP service delivery; and e) preferences related to PPFP and experiences of PPFP access.

Interviews among *husbands* of postpartum women (n = 10) explored issues related to the role of men in PPFP decision-making and community norms related to male engagement in family planning; husbands’ expectations related to early in marriage pregnancy; and their views on community-based provision of postpartum contraception availability.

Interviews among *mothers-in-law* (MILs) (n = 10) of women who had recently given birth focused on family involvement in PPFP decision-making; family dynamics of young couples; norms related to women’s fertility early in marriage and son preference; and their role in family planning and views on community-based provision of PPFP.

Interviews with *frontline health workers* (n = 10) including female auxiliary nurse midwives (ANMs) and ASHAs (accredited social health activists) focused on provision of contraceptive counseling and PPFP care; PPFP and decision-making norms related to postpartum contraceptive uptake; spacing and son preference; myths and misconceptions related to contraception use; feasibility and challenges of delivering community based PPFP services; barriers to implementation and infrastructure challenges related to PPFP service provision.

Interviews among *community leaders* (n = 10), including male and female village panchayat members (local elected officials), NGO workers, and private and government medical officers explored issues related to implementation, cultural factors and the access of PPFP; quality of existing PPFP services; and views related to provision of community-based FP services.

### Data analysis

Throughout data collection, interview data were iteratively assessed for completeness and understanding; probes were refined as needed and assessed for thematic saturation. Interviews were transcribed and translated, and subsequently the transcripts were uploaded onto ATLAS.ti (v. 22) for analysis. A sub-sample of 10 interviews were reviewed to generate a codebook for analysis, which was refined further for code saturation. Two coders reviewed the first 10 interviews independently, and inter-coder agreement reliability was 89% agreement. Data were analyzed by a team of six trained staff members and were regularly reviewed for quality control by lead investigators. Disagreements or conflicts were resolved by the senior author. In case of any issues with the quality of the transcript, the Maharashtra-based scientist assessed quality concerns and discussed with the project team. Lead investigators also performed occasional spot-checks for quality control.

While the study had used Bronfenbrenner’s Ecological Systems Theory in developing the sampling frame and the nature of respondents in the study, this approach was not mirrored in the analysis due to cross-cutting themes that could not be synthesized within one stakeholder group. We did not use a pure grounded theory approach for analysis. However, we used principles of grounded theory to generate codes and guide the development of a framework to understand emerging themes and to understand the social processes and interactions across stakeholder groups. We identified key constructs, particularly reproductive agency, as described by Can-Act-Resist [19] and the role of social norms in driving the emerging themes. We used an inductive approach developed through workshops that synthesized the emergent themes into a framework developed by the study team. A number of inter-connected themes and sub-themes emerged. Coders mapped the themes by constructs as they emerged. Finally, the codes and quotes were organized into five key thematic areas which were synthesized into learnings for FP programming.

### Positionality

The study team comprised a mix of nationalities, ethnicities and genders. The study team was not invested in development of FP programming that promotes women using contraception, but rather our aim is to understand barriers to contraceptive use if desired. The study team was cognizant of the power asymmetries between researchers and the participants, as well as power dynamics in the community that might influence the study. The study team were engaged in detailed planning of stakeholder interviews, and study instrument was designed keeping in mind the constraints faced by the participants such as time. One of the investigators was also a local community expert with decades of experience in both research and project management in the area. With his guidance, the study team followed local protocols and cultural etiquette in the outreach, recruitment, communication and data collection, respecting the priorities and time of the participants. Any discomfort or issue with the interview were brought to the attention of the study team and addressed as swiftly as possible.

## Findings

Participating postpartum women in the study were between 20–31 years of age, while the age of participating husbands ranged from 27 to 42 years. Our research team interviewed five ASHAs, five ANMs and four medical officers (MOs). Local community leaders in the study included four village panchayat leaders and 2 NGO workers; equal representation by males and females was noted in the study.

**Fig 1:**
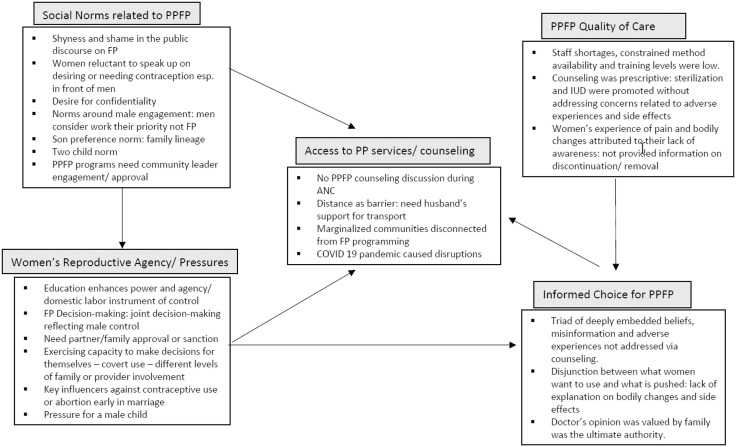
Emerging themes and issues related to postpartum family planning (PPFP) access and use in Maharashtra, India. The figure reflects the themes that emerged through interviews with stakeholders related to postpartum family planning access and use in Maharashtra, India. These themes related to: access to postpartum (PP) services and counseling, women’s reproductive agency and pressures, social norms related to postpartum family planning, postpartum quality of care, and informed choice for post-partum family planning. The discussion points in each of the boxes has been described in the text of the manuscript.

We identified five emergent themes (Fig 1):

### I. *Access to Postpartum Family Planning Services and Counselling*: Few women received PPFP counselling or services, with marginalized communities disproportionately affected by lack of access

Most participants reported that they had not received any information related to PPFP services in their interactions with clinicians during their antenatal care visits or post-delivery.

*No. Family planning was not discussed during their visit. She discussed with me only about investigations such as blood test, weight, BP, urine etc. to be done. Pills were given if they noticed anemia. She came once and then called me and told me where the vaccination will be. (postpartum woman, age 23)*

*I had my all 3 of my deliveries at the primary health center. I visited the primary health center on number of occasions earlier. I used to go once in two months. There was no discussion on family planning as such. (postpartum woman, age 23)*

Only a few participants reported that they received FP information during prenatal care.

*Yes, he said, “If you want to keep spacing between this and the next baby, you can put a CuT (Copper T) in next three months.” There was not much talk, only when he visited to remove the stitches on the tenth day after the delivery. We told him that we will do it (CuT) after three months. (postpartum woman, age 25)*

Health providers noted that reaching marginalized groups was challenging, and tribal and migrant populations were often disconnected from FP programs and from PPFP services.

*There are some special religions or societies such as tribal society or migrant workers, in which even if there were four or five children, they would continue having more. This is due to the fact that information does not reach to them and there is illiteracy. They are taken directly to the hospital at the time of delivery after becoming pregnant. They have no reports. What will you tell them then? The story has not yet reached them. Reaching out to migrants can be difficult at times (medical officer, male, age 36)*.

In some cases, permission or approval of community leaders (the *Sarpanch*) was also needed to seek FP access, with implications for PPFP use.

*Many couples will not participate because they do not know much about it. Sarpanch’s permission has to be sought at the village level. Such a question needs to be discussed. (husband, age 33)*

The COVID-19 pandemic had also disrupted FP access, particularly affecting PPFP services in the community. However, disrupted services were being resumed.

*Last 2 years, there have been no (health) camps. So, what happened in the past has not been very effective. Health services were supposed to reach the grassroots level have not reached or what is being disseminated through social media has not been done as much as it should have been. (village panchayat representative, male, age 38)*

*Earlier we had a primary health care vehicle to take pregnant mothers, but now the vehicle facility has stopped, because of the COVID pandemic. People arrange transport on its own either via four wheelers or two wheelers. (ASHA, age 38)*

### II. *Women’s reproductive agency and pressures*: Postpartum women lacked agency in contraceptive decision-making and often needed their husband’s approval to access PPFP services

Participants viewed education as an important instrument for transformative change that determined contraceptive awareness and communication among couples. Higher education improved knowledge of family planning and elevated women’s ability to convey and convince their families to use PPFP services.

*The problem is that whatever the information is provided to them (women), we don’t know how far this reaches. They will not know the advantages and disadvantages of [contraception]. As she is non- literate, she can’t read about it anywhere. They may also have the influence of their family members assuming that as she is not learned and she does not have capacity to understand. She can’t make that decision. She can’t explain or convince her family members in a good way because she is not well educated. If there is an educated woman in that place, she can come here and read the poster and we explain to her directly or tell her to come to us and we tell her about the method that she would like to adopt. (ANM, age 28)*

However, despite increasing levels of education in the community, women experienced backlash through verbal or physical abuse when they exercised their choice to use FP against the wishes of their family members. In cases where health providers counseled women to access postpartum contraception, couples often believed that their families knew better and eventually the will of their families prevailed.

*If she has any side effects after insertion, household members will blame her and will question her about insertion. In some cases, there can be abuse by family members. If she informs her family members, they will help her when she is in trouble. But if she goes and gets service on her own, then some family members will understand and some may not. (postpartum woman, age 25)*

*We hardly discussed about our contraceptive need and use of family planning with the health care provider. We just asked about how to take care of baby. We have in-laws at home, they have more experience.“(postpartum woman, age 28)*

Family planning decision-making represented a dialogue and often a negotiation for couples. These negotiations led to experiences of pressure relating to use or non-use of contraception, from other family members (usually in-laws).

*“No, it is a couple’s decision meaning husband and wife will discuss first. If we agree with each other, we can take a chance, or if the husband does not think he wants to take a chance, we can discuss our plan with my mother/mother-in-law about family planning and when they would like to take a chance.” (postpartum woman, age 23)*

*Sometimes her husband tells her not to use the CuT (Copper T IUD), then at that time the woman can do nothing. It requires the consent of both partners. But if a woman insists on opposing her husband’s wishes, then there is a quarrel between them. (MIL, age 60)*

Distance was often reported as a barrier preventing PPFP access and role of the husband in overcoming this barrier was critical. Male approval, support for transport and male company was commonly needed by women to reach health services.

*If the distance from home to the hospital is too long, the woman cannot go to the place. At the same time, they may face financial difficulties. It may also be a question of safety, someone will have to accompany her from her home to the hospital. Will the doctors who are there do the proper checkup or treatment when they go to the hospital? They will have to face these difficulties/challenges. (Husband, age 31)*

The desire for a male child continued to be a source of childbearing pressure for couples. This has, in previous research, been known to affect women more than men [36] and can influence the decision-making around spacing contraception, especially when the previous born child was female. Son preference also influenced women’s use of contraception and abortion early in marriage and even led to reports of covert use of contraception. The use of contraception often depended on the sex of the previous child, and if in case the previous birth was a girl, often led to lower use of subsequent contraception. In this scenario, if women did not desire another baby but were unable to voice this desire, they exercised their choice via covertly using contraception. They reported sharing varying degrees of information with their husbands, family members and health workers, depending on whom they considered an ally. These instances of information sharing and hiding were important facets of their exercise of agency and choice, even when the ultimate decision may not have gone their way.

*There is no need of taking permission from anyone in the community. Because it’s up to you to decide whether or not to use family planning. The role of the family may be if it is a boy, but now we have said that when there are girls and if the couple wants to undergo sterilization, they will not get ready easily, so they should be told after the operation that the operation has been done. (postpartum woman, age 31)*

*Yes, they tell us that they want to insert CuT (Copper T IUD). We tell them to come on the fourth or fifth day of menstruation. She will tell some false reason to her family members that she is not feeling well and wants to get some medicines, so she wants to go out and then she visits the health centre and gets the CuT inserted. She also instructs us not to tell her family members regarding her method use. We tell her that we will not disclose. If you come and do not get an IUD then at least start using pills. (ASHA, age 43)*

### III. *Informed choice for postpartum family planning*: Health providers identified misinformation, lack of education, and beliefs in contraceptive myths for lack of informed choice

Health providers participating in our study acknowledged that informed choice was important to enable women’s agency and decision-making, as well as that of their family members. However, if there was a conflict between the provider and the family, the latter prevailed. It was not unexpected or unreasonable for women to consult their family members on PPFP, but it was concerning for health providers when family members superseded women’s choice or desire for PPFP counselling or services. Health providers were particularly concerned about the spread of contraception-related misinformation, especially those related to side effects within women’s social networks as they believed this deterred contraceptive use. In general, providers blamed this misinformation on culturally determined beliefs or on adverse experiences faced by women themselves or someone in their social circle. Providers believed that poor education often led to negative contraceptive beliefs thereby compromising women’s ability to exercise choice, navigate family pressures and participate in PPFP decision-making.

*Yes because, if they want to use contraception, they need to be fully informed about it. If there is no information then it is going to affect another beneficiary. This is because when the CuT (Copper T IUD) is inserted without providing complete information about side effects she might get more bleeding during first few months of menstruation or she may get acidity when she starts taking pills. So, you need to know all these things as it affects other beneficiaries as well. If not, then she goes on spreading wrong information to other women. (ANM, age 28)*

*Yes, there are two or three examples of people who had a hard time after inserting a CuT (Copper T IUD) and removed it immediately within a month. Some people say that they don’t want to insert a CuT after listening to the experiences of others. (medical officer, female, age 32)*

### IV. *Social norms related to postpartum family planning:* While the norms for limiting family size and son preference were strong, contraception was considered a ‘sensitive’ subject and confidentiality was valued

All participants reported having inhibitions about discussing FP with their families including postpartum contraceptive. As a result, women’s ability to initiate conversation about their desire to plan children, use contraception, or negotiate family planning within their families was limited. Postpartum women were reluctant to discuss the subject even with clinicians and valued confidentiality in counseling.

*Women in the villages are shy and they think that other people will come to know about the method they are using. They have these kinds of negative attitudes. Then I tell them that you have had your second and third injection and even if you do not come for collecting condoms, your husband can come and collect. It is available with health workers as well ASHAs and he can collect them from her also. This kind of discussion helps them to reduce their shyness. She sends her husband and he collects the condom. I have such beneficiaries in my center. (ANM, age 57)*

There was a sense that this lack of openness to discussing contraception would affect PPFP programming and limit couples’ participation in counseling or services delivered in public venues or through public platforms (e.g., *panchayat-ghar*). Multiple participant groups indicated that women may feel embarrassed when seeking PPFP counseling or services in front of others.

*Family planning is a very sensitive subject. It can be embarrassing to take the methods if you decide to go through camps or gatherings. If there are a large number of men in a camp, a woman may feel shy to ask for a family planning method. This can also prevent people from attending camps or gatherings. (Husband, age 34)*

### V. *Postpartum Family Planning Quality of Care Issues*: Counseling for PPFP was affected by resource shortages, often tended to be prescriptive, and failed to provide information on side effects of contraceptive methods leading to dissatisfaction with available PPFP health services

Women reported few avenues for PPFP counseling and often relied on alternative sources such as medical stores, community health events, and private providers for counseling and contraception access. In contrast, health providers blamed women’s poor health literacy for delays in PPFP counselling. This often led to low uptake of existing contraception services. Health workers reported that the two-child norm in the community was strong and both influences contraceptive choices and leading to inadvertent consequences such as women hesitating to avail antenatal care services for their third pregnancy.

*Sometimes what happens is that the women do not tell us about their missed period. They come to us four months later after getting pregnant to register for the ANC checkup. They come in time for the first two babies, but if she is pregnant for the third time, she doesn’t tell the ANM or the ASHA worker. They say don’t tell them right away that she is pregnant. While there are injections/pills available, but they are late to avail these services. (ANM, age 43)*

Participants reported that existing counseling availability and quality was limited due to staffing shortages, lack of trained health workers and supply chain issues. Where counselling existed, it was often prescriptive and even coercive instead of being person-centered or focusing on client preferences or goals. The method mix offered to postpartum women usually comprised IUDs and permanent female contraception.

Camps organized for permanent female contraception had limited information on the risks of procedure or side effects. Several women reported dissatisfaction with PPFP services as information on side effects and bodily changes related to contraceptive use and discontinuation (e.g., removal of IUD) was limited. However, health providers attributed women’s dissatisfaction on their poor knowledge rather than the service quality or counseling.

*There was some problem faced by my aunt’s daughter. Her CuT (Copper T IUD) was perforated and she was very upset. Her CuT was inserted at the government hospital. She suffered a lot due to this perforation. (postpartum woman, age 31)*

*They do not know how to use it. After CuT insertion, it might expel which a woman does not understand. If it hurts a little after the CuT insertion, they do not understand it and ask service providers to remove it. They realize that there is bleeding and sometimes the menstruation does not come on time. (medical officer, male, age 36)*

## Discussion

This qualitative study on factors influencing access to and use of PPFP services in India provided a distinct and important lens into multilevel influences on women’s reproductive agency and family planning norms that facilitate or limit postpartum contraceptive use. Our findings reflect challenges faced in accessing PPFP services, especially for the use of reversible contraception methods in the postpartum period, and opportunities for solutions and better care.

Respondents offered perspectives across multiple domains of influence – family (partners and mothers-in-law), community, and health systems (providers) highlighting important common understandings of barriers to PPFP.

*Firstly*, PPFP services were not easily accessible for women due to distance from providers, lack of male partner support, and social norms that limit use of FP until achievement of fertility goals. These access issues were particularly pronounced for marginalized groups.

*Secondly*, Women’s reproductive agency was limited, resulting in constrained decision-making within families. Son preference further influenced women’s fertility and contraceptive choices. Even when women were counseled by health providers, the preferences of their family members, especially the male partner, dominated the decision-making. Some women, however, exercised their agency through covert use of contraception.

*Thirdly*, while health providers placed a premium on informed choice and offering comprehensive contraceptive options, they highlighted that it was difficult to address women’s concerns regarding contraceptive methods available particularly IUDs, due to extensive contraceptive misinformation and fear of side effects. These concerns limited women’s full consideration of contraceptive options in their PPFP decision-making.”

*Fourth*, concerns regarding confidentiality in accessing and receiving services were highlighted, as family planning was considered a ‘sensitive’ subject and was not always supported by family or community for purposes of birth spacing. Consequently, use of group settings for delivery of contraceptives and PPFP counseling was not always feasible.

*Finally*, avenues of high quality PPFP counseling were perceived as limited, partially due to human resource shortages and partially due to concerns that available counseling was coercive rather than person-centered. Lack of adequate information on method discontinuation and inaccurate knowledge gained through women’s networks contributed to women’s dissatisfaction with current PPFP service availability and to low use.

Despite the successes of family planning programs in India, there has been limited attention to family planning needs in the postpartum period. This has primarily been due to the focus on family size limitation, which has led to widespread use of female sterilization as a preferred method of contraception, and less focus on healthy birth spacing. There has been some recognition of the need for better connections between the antenatal services and postpartum care [37], but this desire for a continuum is often not reflected in the service delivery on the ground, likely due to the siloed nature of maternal and family planning service delivery systems and priorities. In another study conducted among five countries, including India, despite over 95% of sampled women reported desire to not have a repeated childbirth within one year of delivery, the use of long-acting spacing contraception remained low [38]. Our study reported that women faced many barriers including physical infrastructure-related barriers of access.

In our study, we found that despite increased levels of women’s education, women continued to experience fertility pressures including that for male children that affected spacing contraception choices. In some cases, women overcame these pressures or used contraception covertly, but in the majority, women opted to adhere to the will of their partners or family members. This finding underscores the importance of social norms in influencing reproductive agency, highlighting the need for family planning programs to enhance women’s reproductive choice while also initiating programs that engage with women’s families and their social networks. Research on women’s agency needs to recognize the diverse manifestations of agency and their implications for FP use, as well as to enhance their application and measurement within FP programs.

This study contributes to a growing body of research on women’s reproductive agency and norms related to PPFP, its conceptualization and how it might predict fertility choices and contraceptive use in the postpartum period [39,40]. While barriers were identified, respondents also highlighted towards ways of improving equal access to quality care. For example, programming that addresses gendered community norms supportive of family planning and expanding these to include reversible contraceptives for use during the postpartum period. Gender-transformative family planning programming is programming that seeks not only to increase access to contraception but also to challenge and shift the underlying gender norms that limit women’s reproductive agency [41]. Such approaches recognize that women’s decisions about fertility and contraceptive use are often shaped by expectations around son preference, unequal power within relationships, and family influence. By actively engaging men, families, and communities, and by promoting equitable decision-making, gender-transformative programs aim to expand women’s autonomy in family planning, and support more inclusive couple communication. Gender transformative family planning interventions could be integrated into postpartum family planning programming in India to address the gendered social norms that were identified as barriers to family planning uptake and use [42,43]. Designing interventions to influence norms related to women’s seeking healthcare postpartum can also enable PPFP access and use. Even as this research is growing, there is a need for rigorous evaluation of interventions in the postpartum period that encourage FP counseling or use [44].

Given the exploratory nature of the study and the focus on systems and access, we were unable to further explore the intersectionality of gender with poverty and ethnicity/caste. However, this intersectionality remains an important focus for future research on contraception use among postpartum women in India.

Our results also showed that the engagement of men in PPFP was critical. Men had influence through many mechanisms, including approval/sanction for PPFP, support for the choices of postpartum women and the provision of instrumental support such as arranging transport or accompanying women to the health center. These accounts of male engagement need further exploration, as some may imply male control or male patronage, rather than agency [45]. Male engagement for PPFP may also vary with contraceptive method type [46]. As research grows in this field, there is a need to examine male engagement models for PPFP, the implications of shifting couple power dynamics inherent in this engagement, and the success of achieving this engagement through modification of gender roles and norms around pleasure and responsibility in intimate relationships and in health seeking. Men as allies have also been instrumental in navigating intergenerational pressures faced by women, especially through negotiations with their mothers in law [47]. A review on mother-in-law influence in FP demonstrates that the grip of MILs on contraceptive access, especially early in marriage, operated through their control of women’s mobility and responsibilities of domestic labour [47]. In this study, MILs reported a decline in their control over their daughter-in-law’s fertility decision-making; despite this, family sanction continued to be important, even overriding the advice of the health providers.

Our findings demonstrate that despite increased modern contraceptive use rates, PPFP continues to be a major gap within FP programs and there is an urgent need to strengthen counseling and examine the FP method mix for PPFP during ANC visits and post-delivery to expand women’s reproductive choice. There is also a need to understand the nature and quality of FP counseling that was available and availed by these women. Our exploratory evidence shows that this cannot be achieved without addressing social barriers that inhibit women’s reproductive agency while accessing PPFP. Nearly all participants reported a reluctance to speak about family planning in public and placed value in confidentiality while seeking FP services. There was discomfort in discussing contraception and sexual life among couples. Greater advocacy is needed to facilitate open and stigma-free discussions on contraceptive use and enable women to express their desire for using contraception, within families and with providers. However, this advocacy must not only be targeted towards women, but include key influencers in their ecosystem who can enable PPFP. One intervention to enable PPFP information and access, including to those women who were not reached during antenatal care is to explore the feasibility of other maternal and child health care delivery systems such as vaccination camps to provide information, counseling and potentially services for PPFP [48]. Other specific interventions may include setting up mobile clinics to ensure access among postpartum women in remote areas, creation of peer circles among women and men to talk about family planning, fertility and contraception-related issues and training of community health workers to understand issues with the uptake of postpartum contraception, in particular postpartum IUD.

While this study addresses an important evidence gap to examine reproductive agency for postpartum contraception, findings must be interpreted in light of the following limitations. The study was a qualitative study and therefore the sample did not allow generalization of findings but allowed us to gain deeper insight into pathways and inter-relationships between different aspects of PPFP agency and norms. Findings of the study were based on perceptions of the diverse stakeholders on PPFP service availability, its delivery and challenges. We were not able to provide any objective accounts of these services through a study of the health services for PPFP care; neither was it our objective to assess the service infrastructure, but only to explore the experiences and perceptions of communities. Our study data utilized self-reports and may be subject to social desirability bias. To overcome this, we provided triangulated accounts of the diverse themes, to the extent possible.

## Conclusion

Our study demonstrates that there is an urgent need to enhance PPFP counseling and services within FP programs in India. These programs must emphasize reproductive choice, broaden the public dialogue on contraceptive use and more actively and meaningfully engage men. Interventions that enhance knowledge and change acceptability of contraception in the community will enhance informed choice for contraceptive use among couples as well as PPFP access and use.

## Supporting information

S1 Questionnaire Inclusivity in global research questionnaire statement. (DOCX)

## References

[1] Dewey KG, Cohen RJ. Does birth spacing affect maternal or child nutritional status? A systematic literature review. Matern Child Nutr. 2007;3(3):151–73. doi: 10.1111/j.1740-8709.2007.00092.x 17539885 PMC6860904

[2] Conde-Agudelo A, Rosas-Bermudez A, Castaño F, Norton MH. Effects of birth spacing on maternal, perinatal, infant, and child health: a systematic review of causal mechanisms. Stud Fam Plann. 2012;43(2):93–114. doi: 10.1111/j.1728-4465.2012.00308.x 23175949

[3] Kannaujiya AK, Kumar K, Upadhyay AK, McDougal L, Raj A, Singh A. Short interpregnancy interval and low birth weight births in India: Evidence from National Family Health Survey 2015-16. SSM Popul Health. 2020;12:100700. doi: 10.1016/j.ssmph.2020.100700 33304985 PMC7710655

[4] Thiel de Bocanegra H, Chang R, Howell M, Darney P. Interpregnancy intervals: impact of postpartum contraceptive effectiveness and coverage. Am J Obstet Gynecol. 2014;210(4):311.e1-311.e8. doi: 10.1016/j.ajog.2013.12.020 24334205

[5] DeFranco EA, Seske LM, Greenberg JM, Muglia LJ. Influence of interpregnancy interval on neonatal morbidity. Am J Obstet Gynecol. 2015;212(3):386.e1-9. doi: 10.1016/j.ajog.2014.11.017 25460837

[6] Conde-Agudelo A, Rosas-Bermúdez A, Kafury-Goeta AC. Birth spacing and risk of adverse perinatal outcomes: a meta-analysis. JAMA. 2006;295(15):1809–23. doi: 10.1001/jama.295.15.1809 16622143

[7] Conde-Agudelo A, Belizán JM. Maternal morbidity and mortality associated with interpregnancy interval: cross sectional study. BMJ. 2000;321(7271):1255–9. doi: 10.1136/bmj.321.7271.1255 11082085 PMC27528

[8] Romano M, Cacciatore A, Giordano R, La Rosa B. Postpartum period: three distinct but continuous phases. J Prenat Med. 2010;4(2):22–5. 22439056 PMC3279173

[9] Kozuki N, Lee ACC, Silveira MF, Victora CG, Adair L, Humphrey J, et al. The associations of birth intervals with small-for-gestational-age, preterm, and neonatal and infant mortality: a meta-analysis. BMC Public Health. 2013;13(Suppl 3):S3. doi: 10.1186/1471-2458-13-S3-S3 24564484 PMC3847557

[10] Dhingra S, Pingali PL. Effects of short birth spacing on birth-order differences in child stunting: Evidence from India. Proc Natl Acad Sci U S A. 2021;118(8):e2017834118. doi: 10.1073/pnas.2017834118 33602815 PMC7923660

[11] Blencowe H, Krasevec J, de Onis M, Black RE, An X, Stevens GA, et al. National, regional, and worldwide estimates of low birthweight in 2015, with trends from 2000: a systematic analysis. Lancet Glob Health. 2019;7(7):e849–60. doi: 10.1016/S2214-109X(18)30565-5 31103470 PMC6560046

[12] Rana MJ, Gautam A, Goli S, Uttamacharya, Reja T, Nanda P, et al. Planning of births and maternal, child health, and nutritional outcomes: recent evidence from India. Public Health. 2019;169:14–25. doi: 10.1016/j.puhe.2018.11.019 30772525 PMC6483972

[13] IIPS. National Family Health Survey (NFHS-5) 2019-21: India: Volume I. Mumbai, India: IIPS & ICF; 2021.

[14] Suri S. India’s Family Planning Mission Puts Burden of Sterilisation on Women at the Cost of Their Health. 2022. Available from:https://www.orfonline.org/research/burden-of-sterilisation-on-women-at-the-cost-of-their-health/

[15] Rajan S, Speizer IS, Calhoun LM, Nanda P. Counseling during Maternal and Infant Health Visits and Postpartum Contraceptive use in Uttar Pradesh, India. Int Perspect Sex Reprod Health. 2016;42(4):167–78. doi: 10.1363/42e2816 28649295 PMC5477656

[16] Track 20. Trends in the Uptake of Postpartum Family Planning. [cited July 1, 2023]. Available from: https://www.track20.org/pages/data_analysis/in_depth/PPFP/trends.php

[17] Rashmi K, Renuka T. Misconceptions and Myths About Intrauterine Contraceptive Devices and Their Impact on Usage. RGUHS J Med Sci. 2025;15(3).

[18] Upadhyay UD, Dworkin SL, Weitz TA, Foster DG. Development and validation of a reproductive autonomy scale. Stud Fam Plann. 2014;45(1):19–41. doi: 10.1111/j.1728-4465.2014.00374.x 24615573

[19] Raj A, Dey A, Rao N, Yore J, McDougal L, Bhan N, et al. The EMERGE framework to measure empowerment for health and development. Soc Sci Med. 2024;351(Suppl 1):116879. doi: 10.1016/j.socscimed.2024.116879 38825382

[20] Bhan N, Raj A. From choice to agency in family planning services. Lancet. 2021;398(10295):99–101. doi: 10.1016/S0140-6736(21)00990-9 33971154

[21] Singh A, Kumar K, Yadav AK, James KS, McDougal L, Atmavilas Y, et al. Factors Influencing the Sex Ratio at Birth in India: A New Analysis based on Births Occurring between 2005 and 2016. Stud Fam Plann. 2021;52(1):41–58. doi: 10.1111/sifp.12147 33616232 PMC8049007

[22] Pörtner CC. Birth Spacing and Fertility in the Presence of Son Preference and Sex-Selective Abortions: India’s Experience Over Four Decades. Demography. 2022;59(1):61–88. doi: 10.1215/00703370-9580703 34779484

[23] Annual Report: Ministry of Health and Family Welfare, Government of India. 2016.

[24] Tully KP, Stuebe AM, Verbiest SB. The fourth trimester: a critical transition period with unmet maternal health needs. Am J Obstet Gynecol. 2017;217(1):37–41. doi: 10.1016/j.ajog.2017.03.032 28390671

[25] Cheng C-Y, Fowles ER, Walker LO. Continuing education module: postpartum maternal health care in the United States: a critical review. J Perinat Educ. 2006;15(3):34–42. doi: 10.1624/105812406X119002 17541458 PMC1595301

[26] Coomson JI, Manu A. Determinants of modern contraceptive use among postpartum women in two health facilities in urban Ghana: a cross-sectional study. Contracept Reprod Med. 2019;4:17. doi: 10.1186/s40834-019-0098-9 31645994 PMC6802318

[27] Wassihun B, Wosen K, Getie A, Belay K, Tesfaye R, Tadesse T, et al. Prevalence of postpartum family planning utilization and associated factors among postpartum mothers in Arba Minch town, South Ethiopia. Contracept Reprod Med. 2021;6(1):6. doi: 10.1186/s40834-021-00150-z 33648557 PMC7923452

[28] Abraha TH, Gebrezgiabher BB, Aregawi BG, Belay DS, Tikue LT, Welay GM. Predictors of postpartum contraceptive use in rural Tigray region, northern Ethiopia: a multilevel analysis. BMC Public Health. 2018;18(1):1017. doi: 10.1186/s12889-018-5941-4 30115045 PMC6097291

[29] Adams YJ, Smith BA. Integrative Review of Factors That Affect the Use of Postpartum Care Services in Developing Countries. J Obstet Gynecol Neonatal Nurs. 2018;47(3):371–84. doi: 10.1016/j.jogn.2018.02.006 29524378

[30] Mihretie GN, Getie SA, Shiferaw S, Ayele AD, Liyeh TM, Kassa BG, et al. Interbirth interval practices among reproductive age women in rural and Urban kebeles in Farta Woreda: Case-control study. PLoS One. 2022;17(1):e0256193. doi: 10.1371/journal.pone.0256193 35085250 PMC8794163

[31] Wuni C, Turpin CA, Dassah ET. Determinants of contraceptive use and future contraceptive intentions of women attending child welfare clinics in urban Ghana. BMC Public Health. 2017;18(1):79. doi: 10.1186/s12889-017-4641-9 28764670 PMC5539629

[32] Keesara S, Juma PA, Harper CC, Newmann SJ. Barriers to postpartum contraception: differences among women based on parity and future fertility desires. Cult Health Sex. 2018;20(3):247–61. doi: 10.1080/13691058.2017.1340669 28705100

[33] Kayi EA, Biney AAE, Dodoo ND, Ofori CAE, Dodoo FN-A. Women’s post-abortion contraceptive use: Are predictors the same for immediate and future uptake of contraception? Evidence from Ghana. PLoS One. 2021;16(12):e0261005. doi: 10.1371/journal.pone.0261005 34932576 PMC8691597

[34] IIPS. National Family Health Survey (NFHS-4). India Factsheet. Mumbai: International Institute for Population Sciences; 2015. Available from: http://rchiips.org/NFHS/factsheet_NFHS-4.shtml

[35] Bronfenbrenner U. Toward an experimental ecology of human development. Am Psychol. 1977;32(7):513–31. doi: 10.1037/0003-066x.32.7.513

[36] Bhan N, Johns NE, Chatterji S, Thomas EE, Rao N, Ghule M, et al. Validation of the Fertility Norms Scale and Association with Fertility Intention and Contraceptive Use in India. Stud Fam Plann. 2023;54(1):39–61. doi: 10.1111/sifp.12227 36691257 PMC11147959

[37] Achyut P, Mishra A, Montana L, Sengupta R, Calhoun LM, Nanda P. Integration of family planning with maternal health services: an opportunity to increase postpartum modern contraceptive use in urban Uttar Pradesh, India. J Fam Plann Reprod Health Care. 2016;42(2):107–15. doi: 10.1136/jfprhc-2015-101271 26622056 PMC4853573

[38] Pasha O, Goudar SS, Patel A, Garces A, Esamai F, Chomba E, et al. Postpartum contraceptive use and unmet need for family planning in five low-income countries. Reprod Health. 2015;12(Suppl 2):S11. doi: 10.1186/1742-4755-12-S2-S11 26063346 PMC4464604

[39] Bhan N, Thomas E, Dixit A, et al. Measuring women’s agency and gender norms in family planning: What do we know and where do we go? Center on Gender Equity and Health (GEH); 2020.

[40] McCleary-Sills J, McGonagle A, Malhotra A. Women’s demand for reproductive control. Understanding and addressing gender barriers. Washington, DC: International Center for Research on Women; 2012.

[41] Jacobson JL. Transforming family planning programmes: towards a framework for advancing the reproductive rights agenda. Reprod Health Matters. 2000;8(15):21–32. doi: 10.1016/s0968-8080(00)90003-x 11424265

[42] Raj A, Ghule M, Johns NE, Battala M, Begum S, Dixit A, et al. Evaluation of a gender synchronized family planning intervention for married couples in rural India: The CHARM2 cluster randomized control trial. EClinicalMedicine. 2022;45:101334. doi: 10.1016/j.eclinm.2022.101334 35274093 PMC8902598

[43] Averbach S, Johns NE, Ghule M, Dixit A, Begum S, Battala M, et al. Understanding quality of contraceptive counseling in the CHARM2 gender-equity focused family planning intervention: Findings from a cluster randomized controlled trial among couples in rural India. Contraception. 2023;118:109907. doi: 10.1016/j.contraception.2022.10.009 36328094 PMC10695301

[44] Cleland J, Shah IH, Daniele M. Interventions to Improve Postpartum Family Planning in Low- and Middle-Income Countries: Program Implications and Research Priorities. Stud Fam Plann. 2015;46(4):423–41. doi: 10.1111/j.1728-4465.2015.00041.x 26643491

[45] Nazarbegian M, Averbach S, Johns NE, Ghule M, Silverman J, Lundgren R, et al. Associations between Contraceptive Decision-Making and Marital Contraceptive Communication and use in Rural Maharashtra, India. Stud Fam Plann. 2022;53(4):617–37. doi: 10.1111/sifp.12214 36193029 PMC10695302

[46] McDougal L, Silverman JG, Singh A, Raj A. Exploring the relationship between spousal violence during pregnancy and subsequent postpartum spacing contraception among first-time mothers in India. EClinicalMedicine. 2020;23:100414. doi: 10.1016/j.eclinm.2020.100414 32639480 PMC7329749

[47] Bhan N, Sodhi C, Achyut P, Thomas EE, Gautam A, Raj A. Mother-in-law’s influence on family planning decision-making and contraceptive use: A review of evidence. Center on Gender Equity and Health, UC San Diego; 2022.

[48] Averbach S, Thomas EE, Kully G, Nazarbegian M, Ghule M, Rabin BA, et al. Understanding feasibility and acceptability of implementation of linking delivery of family planning and infant vaccination care in rural Maharashtra, India: a qualitative study. BMC Pregnancy Childbirth. 2023;23(1):519. doi: 10.1186/s12884-023-05830-z 37454051 PMC10349507

